# LncRNA MALAT1 Aggravates Renal Tubular Injury *via* Activating LIN28A and the Nox4/AMPK/mTOR Signaling Axis in Diabetic Nephropathy

**DOI:** 10.3389/fendo.2022.895360

**Published:** 2022-06-23

**Authors:** Panai Song, Yinyin Chen, Zhiwen Liu, Hong Liu, Li Xiao, Lin Sun, Jiali Wei, Liyu He

**Affiliations:** ^1^ Department of Nephrology, The Second Xiangya Hospital, Central South University, Changsha, Hunan, China; ^2^ Hunan Key Laboratory of Kidney Disease and Blood Purification, Changsha, Hunan, China; ^3^ Department of Nephrology, Hunan Provincial People’s Hospital, Changsha, China; ^4^ Department of Nephrology, Hainan General Hospital, Haiko, China

**Keywords:** LIN28A, diabetic nephropathy, renal tubular injury, AMPK/mTOR signaling, MALAT1

## Abstract

**Background:**

Diabetic nephropathy (DN) is a serious complication among patients with diabetes. Elucidating its pathogenesis is crucial for identifying novel biomarkers and therapeutic targets for DN.

**Methods:**

DN tissues were harvested for examining MALAT1, LIN28A and Nox4. Human kidney-2 (HK-2) cells were treated with high glucose (HG) for establishing a cell model of DN. Cell viability was examined by MTT assay. HG-induced cell apoptosis and secretion of TNF-α and IL-6 were analyzed by TUNEL and ELISA assays, respectively. RIP and RNA pull-down assays were applied to analyze the interaction between MALAT1, LIN28A and Nox4 in HK-2 and human embryonic kidney 293T (HEK-293T) cells. A rat model of DN was established to determine the role of MALAT1 in DN *in vivo*.

**Results:**

MALAT1, LIN28A and Nox4 were upregulated in DN tissues and HG-treated HK-2 cells. Overexpression of MALAT1, LIN28A or Nox4 reduced cell viability and enhanced cell apoptosis, ROS generation and secretion of inflammatory cytokines in HG-treated HK-2 cells, whereas knockdown of MALAT1, LIN28A or Nox4 exerted opposite effects. Furthermore, MALAT1 directly interacted with LIN28A. Moreover, MALAT1 facilitated the interaction between LIN28A and Nox4 to increase Nox4 stability. Knockdown of Nox4 relieved HG-induced injury by suppressing the AMPK/mTOR signaling in HK-2 cells. Knockdown of MALAT1 alleviated renal tubular epithelial injury by suppressing LIN28A and the Nox4/AMPK/TOR signaling in DN.

**Conclusion:**

MALAT1 activates the AMPK/mTOR signaling *via* interacting with LIN28A to stabilize Nox4 mRNA, thereby aggravating high glucose-induced renal tubular epithelial injury. Our findings provide potential therapeutic targets for DN.

## Highlight

1. MALAT1, LIN28A and Nox4 are upregulated in diabetic nephropathy tissues and high glucose-treated HK-2 cells.
2. MALAT1 directly interacts with LIN28A.
3. LIN28A targets Nox4 to enhance the stability of Nox4 mRNA.
4. Nox4 aggravates high glucose-induced injury *via* activating the AMPK/mTOR signaling.
5. MALAT1 aggravates renal tubular epithelial injury by interacting with LIN28A to activating the Nox4/AMPK/mTOR signaling in rats with diabetic nephropathy.

## Introduction

Diabetic nephropathy (DN) is a very common complication of diabetes and affects millions of diabetic patients each year worldwide (1). DN progressively causes chronic kidney damage and is the leading cause of end-stage renal disease (ESRD) (2, 3). In DN, renal vessels, tubules and glomeruli were disrupted, leading to impaired kidney functions and eventually ESRD (4). Although DN has been previously considered as a glomerular disorder, renal tubular injury starts in the early stage of DN and accelerates its progression (5). The degree of tubular injury is an important determinant of DN prognosis, and early diagnosis and treatment of tubular injury might prevent DN and delay its progression. In the context of diabetes, due to metabolic disorders, inflammation, urine composition and hemodynamic change, renal tubular epithelial cells exhibit oxidative stress and produce various inflammatory cytokines, resulting in interstitial inflammation and fibrogenesis and promoting the onset and development of DN (4). Therefore, elucidating the pathogenesis of renal tubular injury is of great significance to identify novel drug targets for DN treatment.

Long non-coding RNAs (lncRNAs) are key regulators in DN (6, 7). For instance, Ge et al. reported that lncRNA NR_038323 alleviated renal fibrosis *via* sponging miR-324-3p and inactivating p38MAPK and ERK1/2 pathways (8). LncRNA SOX2OT regulated the Akt/mTOR-mediated autophagy to ameliorate renal injury in DN (9). A well-studied lncRNA metastasis associated lung adenocarcinoma transcript 1 (MALAT1) exerts crucial functions in numerous disorders such as cancers (10). Intriguingly, MALAT1 is highly expressed in DN and high glucose-induced HK-2 cells and enhances renal fibrosis in DN (11). Moreover, knockdown of MALAT1 improved kidney functions after duodenal-jejunal bypass surgery in diabetic rats (12). Although growing advances have been made, the role of MALAT1 in the regulation of renal tubular epithelial injury in DN and underlying regulatory mechanism are still largely unknown.

As an RNA binding protein, LIN28 paralogs (LIN28A and LIN28B) directly interacts with target RNAs to maintain their stability, which serves key roles in proliferation, development and pluripotency (13, 14). Recently, emerging evidence has revealed the implication of LIN28 in kidney diseases. Jung and colleagues reported that LIN28A repressed TGF-β-induced renal fibrosis *via* restraining SMAD3 activity (15). MiR-379-5p alleviated renal fibrosis in DN *via* targeting the LIN28B/let-7 axis (16). However, the role of LIN28A in DN, especially in renal tubular epithelial injury, has not been reported. In addition, the interaction between MALAT1 and LIN28A has been identified in osteosarcoma cells (17), suggesting potential association of MALAT1 and LIN28A in DN.

NADPH oxidase (Nox) is the major source of cellular ROS (18), which contributes to various kidney diseases. As the major Nox in the kidney, Nox4 generates H_2_O_2_ which modulates kidney functions. Importantly, Nox4 are implicated in various kidney diseases including DN through activation of multiple signaling (19). Nox4 has been reported to be upregulated in high glucose-treated renal tubular epithelial cells, and it regulated high glucose-induced cell apoptosis through the Notch signaling (20). The Nox4/AMPK/mTOR signaling play important roles in diabetic renal disease (21). Puerarin ameliorated diabetic renal injury by inhibiting the expression of Nox4 in podocytes, indicating that Nox4 could be targeted for DN treatment. Therefore, it is essential to elucidate the underlying mechanism by which Nox4 is involved in high glucose-induced renal tubular epithelial cell injury. Besides, whether LIN28A maintains the stability of Nox4 has never been reported.

Taken together, we hypothesized that MALAT1 might enhance renal tubular epithelial injury in DN through interacting with LIN28A and the Nox4/AMPK/mTOR signaling. Here, we firstly demonstrated that MALAT1 interacted with LIN28A and facilitated LIN28A-mediated maintenance of Nox4 stability to activate the AMPK/mTOR signaling, thereby aggravating renal tubular injury in DN. Our study not only sheds lights on the pathogenesis of DN, but also provides therapeutic targets for DN treatment.

## Methods

### Clinical Specimens

Kidney specimens were collected from patients with DN or minimal change disease (Normal) who were diagnosed at The Second Xiangya Hospital of Central South University and used for subsequent RNA extraction and immunohistochemistry (IHC) staining. Patients aged ≥18 years with nephropathy due to diabetes type 1 or type 2 were included in this study and provided written informed consent. These patients did not receive any treatment previously. Patients with other diseases, such as other rental diseases, autoimmune diseases, cancers and cardiovascular diseases were excluded. Our study got approval from the Ethics Committee of The Second Xiangya Hospital of Central South University.

### Cell Culture and Treatment

Human renal tubular epithelial HK-2 cells and HEK-293T cells were provided by the Cell Bank of Chinese Academy Sciences (Shanghai, China). HK-2 cells were treated with normal glucose (NG, 5.5 mM), high glucose (HG, 30.5 mM) or the osmotic control high mannitol (HM, 5.5 mM glucose and 25 mM mannitol) for 24, 48 or 72 h (22), which were used for subsequent analysis of HG-induced cell injury. Glucose (G7021) and mannitol (M4125) were obtained from Sigma (St. Louis, MO, USA). For 5-aminoimidazole-4-carboxamide ribonucleotide (AICAR) or propranolol treatment, cells were treated with AICAR (S1802, Selleck, Houston, TX, USA) at 1 mM or propranolol (S4076, Selleck) at 100 µM.

### Cell Transfection

MALAT1 and coding sequences of LIN28A and Nox4 were cloned into the pcDNA3.1 vector (V79020, ThermoFisher, Waltham, MA, USA) for overexpression of MALAT1 (oe-MALAT1), LIN28A (oe-LIN28A) and Nox4 (oe-Nox4). siRNA (10 nM) was selected for transient transfection thanks to its easy generation and introduction into cells with high efficiency. Specially, si-RNAs against MALAT1 (si-MALAT1), LIN28A (si-LIN28A) and Nox4 (si-Nox4) and scrambled negative control siRNAs (si-NC) were purchased from RiboBio (Guangzhou, China). HK-2 cells were transfected with vector, oe-MALAT1, si-MALAT1, oe-LIN28A, si-LIN28A, oe-Nox4, si-Nox4, si-LIN28A+vector, si-LIN28A+oe-Nox4, si-MALAT1+vector, si-MALAT1+oe-LIN28A or si-NC using the Lipo3000 reagent (L3000015, ThermoFisher) following the manual. Cells were harvested at 48 hours after transfection for subsequent assays. In some assays, cells were harvested and treated with NG or HG. For *in vivo* knockdown of MALAT1, viral vector-based shRNA with high transduction efficiency and long-term effect was used. Specially, the shRNA against MALAT1 (sh-MALAT1, Sigma) and scrambled shRNA (sh-NC, Sigma) were inserted into the pLKO.1 lentiviral vector (10878, Addgene, Watertown, MA, USA), sh-MALAT1 and sh-NC lentiviral particles were packaged in HEK-293T cells.

### A Rat Model of DN

Sprague-Dawley male healthy rats (6-8-week-old) were provided by Hunan SJA laboratory animal Co., LTD (Changsha, Hunan, China) and blindly divided in to 4 groups: control, DN, DN+sh-NC and DN+sh-MALAT1. In the control group, rats were fed normally. In DN groups, rats were fed a high-glucose and fat diet for 8 weeks and intraperitoneally injected with streptozotocin (STZ, S0130, Sigma) at 55 mg/kg. Subsequently, the blood glucose was monitored every 3 days. DN was considered to be successfully established in rats which met following criteria: blood glucose > 16.7 mmol/L and 24 h urine volume and protein increased 150% after two weeks. DN rats were intravenously injected with 2×10^7^ sh-NC or sh-MALAT1 lentiviral particles and fed a high-glucose and fat diet for 5 months. Finally, kidneys were harvested for subsequent assays. Animal procedures were approved by The Animal Care and Use Committee of The Second Xiangya Hospital of Central South University.

### Real-Time Quantitative Reverse-Transcription PCR (qRT-PCR)

Tissues from patients and rats were homogenized. Total RNA was extracted from HK-2 cells and tissue homogenates using Trizol from Beyotime (R0016, Shanghai, China). Subsequently, RNA was quantified and reversely transcribed into cDNA. The expression of MALAT1, LIN28A and Nox4 was determined using quantitative PCR with SYBR Green (QPK-201, TOYOBO, Tokyo, Japan) and normalized to GAPDH. Gene expression was calculated using the 2^−ΔΔCt^ method. Primers were listed in **Table 1**.

**Table 1:** qRT-PCR primers.

Human MALAT1	5’-GAGTTCTAATTCTTTTTACTGCTCAATC-3’
	5’-TCAAGTGCCAGCAGACAGCA-3’
Rat MALAT1	TGCAGTGTGCCAATGTTTCG
	GGCCAGCTGCAAACATTCAA
Human LIN28A	5’-TTGTCTTCTACCCTGCCCTCT-3’
	5’-GAACAAGGGATGGAGGGTTTT-3’
Human Nox4	5’-CCGGCTGCATCAGTCTTAACC-3’
	5’-TCGGCACAGTACAGGCACAA-3’
Human GAPDH	5’-CTCTGCCCCCTCTGCTGAT-3’
	5’-GTGCAGGAGGCATTGCTGAT-3’
Rat GAPDH	5’- ATGACTCTACCCACGGCAAG-3’
	5’- CTGGAAGATGGTGATGGGTT-3’

### mRNA Stability Analysis in Response to Actinomycin D Treatment

Actinomycin D, a widely used RNA transcription inhibitor for shutting off mRNA transcription, was used to investigate the decay rates of endogenous mRNAs. Briefly, HK-2 and HEK-293T cells were treated with actinomycin D (S8964, Selleck) at 5 µg/mL for indicated time. Subsequently, RNA was extracted and reversely transcribed into cDNA. The abundance of MALAT1 and Nox4 mRNA were analyzed by qRT-PCR.

### MTT Assay for Cell Viability

The culture medium was removed. 100 µL of fresh culture medium and 10 µL of MTT (M6494, ThermoFisher) were mixed well and added into each well. Cells were then incubated for 4 hours, and 50 µL of DMSO was added, and the absorbance (490 nm) was measured.

### Hematoxylin and Eosin (H&E), Masson and Immunohistochemistry (IHC) Staining

Kidney specimens from patients and rats were fixed, embedded and sliced into 5-µm sections. Slices were subsequently deparaffinized and rehydrated. H&E and Masson’s trichrome staining were applied to analyze renal histological change and collagen deposition. For IHC staining, antigen was retrieved in antigen retrieval solution, and slices were incubated with anti-LIN28A (1:50, ab175352, Abcam, Cambridge, UK) or anti-Nox4 (1:100, ab133303, Abcam). Subsequently, slices were incubated with an HRP-conjugated secondary antibody for 1 h. DAB (P0203, Beyotime) was used to visualize the signal. Slices were then stained with hematoxylin, mounted and imaged with a BX51 microscope from Olympus (Tokyo, Japan).

### Terminal Deoxynucleotidyl Transferase dUTP Nick End Labeling (TUNEL) Assay

The Click-iT TUNEL Assay kit (C10337) was obtained from ThermoFisher and used for analyzing cell apoptosis. HK-2 cells were fixed and permeabilized in 0.2% Triton X-100. Kidney tissues from rats were embedded in paraffin and sliced into 5-µm sections. Slices were subsequently deparaffinized, fixed and permeabilized in 0.2% Triton X-100. TdT rection was performed in cells and slices, and the Click-iT reaction was performed for detecting apoptotic cells. Cells and slices were stained with DAPI (C1005, Beyotime), mounted and imaged under a Leica confocal microscope (Wetzlar, Germany).

### Enzyme-Linked Immunosorbent Assay (ELISA)

The culture supernatants of HK-2 cells and rat serum were harvested and stored at -80˚C until use. The concentrations of tumor necrosis factor-α (TNF-α), interleukin-6 (IL-6) and albumin were determined by ELISA kits. Human and rat TNF-α (ab181421 and ab108913), IL-6 (ab178013 and ab100772) and albumin (ab108788 and ab108789) ELISA kits were obtained from Abcam.

### Measurement of ROS

HK-2 cells were stained with using the fluorescent probes dihydroethidium (DHE; Invitrogen, Carlsbad, CA, MA) at 5 µM for 30 min at 37°C in the dark. DHE can be oxidized and then intercalates within DNA, staining the nucleus a bright fluorescent red. Subsequently, cells were washed with PBS for 10 min. Then, Intracellular ROS levels were measured using a fluorometer with excitation at 535 nm and emission at 610 nm (Varioskan™ LUX Multimode Microplate Reader; Thermo Fisher Scientific Inc.). Images were acquired at room temperature and DHE fluorescence intensity was measured using the ImageJ software (NIH, Bethesda, MD).

### Biochemical Analysis

A drop of blood was collected from rat tails and the level of blood glucose was examined using the AlphaTRAK glucose meter (Zoetis, Parsippany, NJ, USA) following the manufacturer’s recommendation. Serum was prepared and urine was collected. The concentrations of blood urea nitrogen (BUN), serum and urinary creatinine were measured using the BUN colorimetric detection kit (EIABUN, ThermoFisher) and creatinine assay kit (ab65340, Abcam), respectively.

### RNA Immunoprecipitation (RIP)

HK-2 and HEK-293T cells were harvested and resuspend in nuclear isolation buffer (20 mM MgCl2, 40 mM Tris-HCl, 1.28 M sucrose and 4% Triton X-100). The nuclei were pelleted by centrifugation and resuspended in RIP buffer (5 mM EDTA, 0.5 mM DTT, 25 mM Tris, 150 mM KCl, 0.5% NP40, RNase and protease inhibitors). Chromatin was sheared, and the supernatants were collected by centrifugation. The anti-LIN28A (ab175352) and normal IgG control (ab37415, Abcam) were pre-coated on magnetic beads, mixed with the supernatants and incubated overnight. Subsequently, immunoprecipitated RNA was recovered using TRIzol, and the abundance of MALAT1 and Nox4 mRNA were analyzed by qRT-PCR.

### RNA-Protein Pull-Down Assay

HK-2 and HEK-293T cells were lysed, and cell lysates were harvested. Biotinylated MALAT1 or Nox4 RNAs (RiboBio) were added into cell lysates and incubated for 5 h at 4 °C for forming RNA-Protein complexes. Subsequently, streptavidin-conjugated magnetic beads (88816, ThermoFisher) were added, and samples were incubated for 2 h with gentle rotation at 4 °C. The RNA-Protein complexes were pulled down by magnetic beads and eluted for western blotting analysis of LIN28A.

### Western Blotting

Rats were sacrificed, and kidneys were excised and weighed. Parts of kidneys were homogenized in lysis buffer. The supernatants of tissue homogenates and cell lysates were collected and quantified using the BCA kit (ab102536, Abcam). Protein (20 µg) was loaded each lane and electrophoresed prior to transfer to PVDF membranes (88518, ThermoFisher). Membranes were then blocked and incubated with anti-LIN28A (1:500, ab279647), anti-Nox4 (1:1000, ab133303), anti-AMPK (1:2000, ab80039), anti-p-AMPK (1:1000, 2531), anti-mTOR (1:1000, ab2732), anti-p-mTOR (1:500, 2971), anti-Bcl-2 (1:1000, ab196495), anti-Bax (1:500, ab32503) and anti-β-actin (1:6000, ab8227) overnight. Next day, membranes were incubated with an HRP-conjugated secondary antibody for 1 h. The bands were visualized using ECL substrate (1705061) from Bio-Rad (Hercules, CA, USA) and analyzed using the Image J software. Antibodies were from Abcam and Cell Signaling Technology (Danvers, MA, USA).

### Statistical Analysis

Data from at least three independent assays were expressed as mean ± standard deviation. The variance in two and multiple groups were analyzed by the Student’s *t* test and one-way analysis of variance (ANOVA), respectively. *P*<0.05 was statistically significant. *P<0.05, **P<0.01 and ***P<0.001.

## Results

### MALAT1 Aggravated High Glucose-Induced Injury in HK-2 Cells

**Figure 1:**
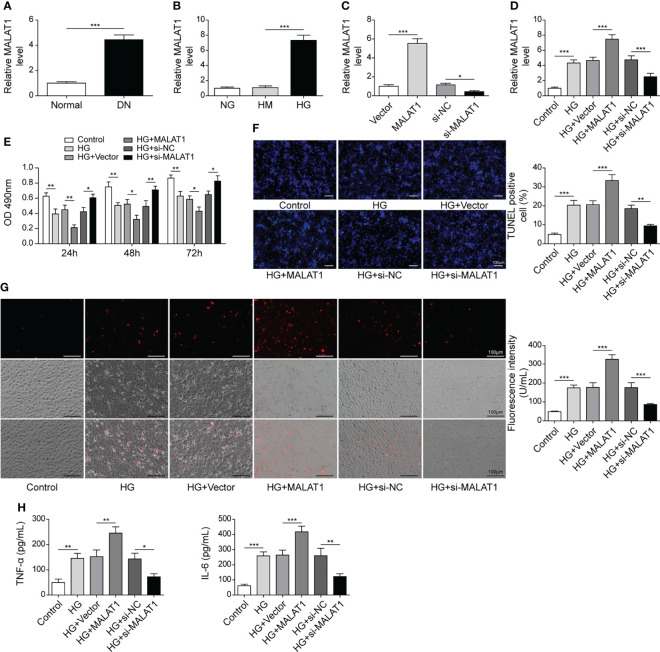
MALAT1 aggravated HG-induced injury in HK-2 cells. **(A)** qRT-PCR analysis of MALAT1 in DN and normal tissues (n=6). **(B)** qRT-PCR analysis of MALAT1 in HK-2 cells with NG, HM or HG (n=4). **(C)** The expression of MALAT1 was validated using qRT-PCR (n=4). HK-2 cells were transfected with vector, oe-MALAT1, si-NC or si-MALAT1 and subsequently treated with HG for 48 h **(D)** qRT-PCR analysis of MALAT1 (n=4). **(E)** Cell viability was examined by MTT assays (n=4). **(F)** TUNEL staining (TUNEL, green. Scale bar=100 µM) were examined. **(G)** Cellular ROS levels were examined by a fluorometric ROS sensor (red, n=4). **(H)** Secretion of TNF-α and IL-6 into culture supernatants (n=4). *P < 0.05, **P < 0.01 and ***P < 0.001.

To explore the roles of MALAT1 in DN, we analyzed its expression and found that DN tissues showed increased MALAT1 expression (**Figure 1A**). Subsequently, we examined MALAT1 expression in HK-2 cells treated with normal glucose (NG), high mannitol (HM) or high glucose (HG). Compared to NG and HM, HG significantly enhanced MALAT1 expression (**Figure 1B**). Moreover, MALAT1 was overexpressed by transfection of oe-MALAT1 and silenced by si-MALAT1 (**Figure 1C**). Our results showed that HG further enhanced MALAT1 expression in cells transfected with oe-MALAT1 but failed to induce MALAT1 expression in cells transfected with si-MALAT1 (**Figure 1D**). Compared to NG, HG reduced HK-2 cell viability (**Figure 1E**). Overexpression of MALAT1 accelerated the reduction of cell viability, whereas silencing of MALAT1 maintained cell viability in response to HG treatment (**Figure 1E**). Furthermore, we found that HG induced cell apoptosis, ROS generation and the secretion of TNF-α and IL-6, which were further enhanced by overexpression of MALAT1 but suppressed by knockdown of MALAT1 (**Figures 1F–H**). Our findings demonstrated that MALAT1 enhanced HK-2 cell apoptosis, ROS generation and inflammatory cytokine secretion in response to HG treatment.

### LIN28A Enhanced HG-Induced Cell Apoptosis, ROS Generation and Inflammatory Cytokine Secretion

**Figure 2:**
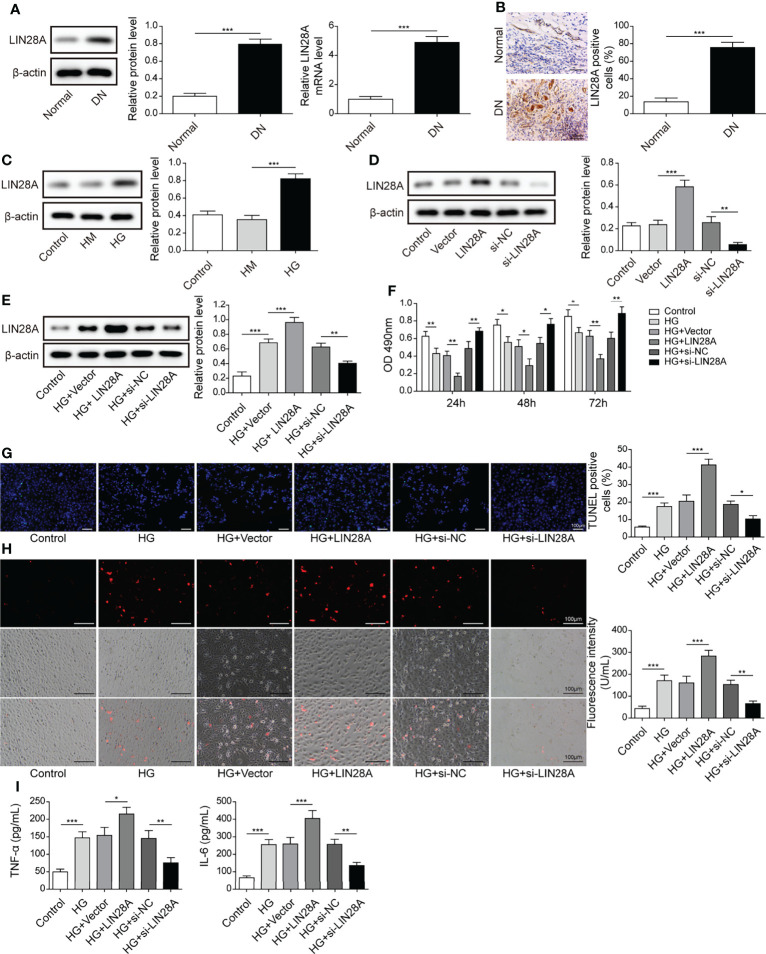
LIN28A enhanced HG-induced cell apoptosis, ROS generation and inflammatory cytokine secretion. **(A)** The expression of LIN28A in DN and normal tissues was analyzed by qRT-PCR and western blotting (n=6). **(B)** IHC staining of LIN28A. **(C)** Protein levels of LIN28A in HK-2 cells treated with NG, HM or HG were examined by western blotting (n=4). **(D)** Western blotting analysis of LIN28A in HK-2 cells (n=4). HK-2 cells were transfected with vector, oe-LIN28A, si-NC or si-LIN28A and subsequently treated with HG for 48 h **(E)** LIN28A was detected using western blotting (n=4). **(F)** Cell viability was examined by MTT assays (n=4). **(G)** TUNEL staining (TUNEL, green. Scale bar=100 µM) were examined. **(H)** Cellular ROS levels were examined by a fluorometric ROS sensor (red, n=4. Scale bar=100 µM). **(I)** Secretion of TNF-α and IL-6 into culture supernatants (n=4). *P< 0.05, **P< 0.01 and ***P< 0.001.

As the expression of LIN28A and MALAT1 have been reported to be positively correlated (17), we examined LIN28A expression in DN tissues. Elevated LIN28A expression was validated by qRT-PCR, western blotting and IHC staining (**Figures 2A, B**). Moreover, HG treatment upregulated LIN28A in HK-2 cells (**Figure 2C**). LIN28A was overexpressed by transfection of oe-LIN28A and knocked down by transfection of si-LIN28A (**Figure 2D**). HG-induced LIN28A expression was further promoted by transfection of oe-LIN28A in HK-2 cells but inhibited by transfection of si-LIN28A (**Figure 2E**). Overexpression of LIN28A obviously impaired HK-2 cell viability and promoted cell apoptosis in response to HG treatment, whereas knockdown of LIN28A significantly weakened these effects (**Figures 2F, G**). Besides, HG-induced ROS generation was strengthened by overexpression of LIN28A but reduced by silencing of LIN28A in HK-2 cells (**Figure 2H**). Also, overexpression of LIN28A enhanced HG-induced secretion of TNF-α and IL-6 in HK-2 cells, but knockdown of LIN28A significantly reduced their secretion (**Figure 2I**). Collectively, these results demonstrated that LIN28A promoted HG-induced injury in HK-2 cells.

### MALAT1 Interacted With LIN28A in HK-2 Cells

**Figure 3:**
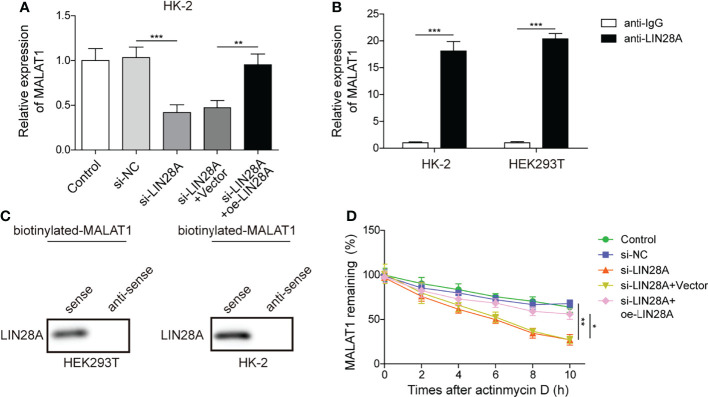
MALAT1 interacted with LIN28A in HK-2 cells. **(A)** qRT-PCR analysis of MALAT1 in HK-2 cells transfected with si-NC, si-LIN28A, si-LIN28A + vector or si-LIN28A + oe-LIN28A (n=4). **(B)** The enrichment of MALAT1 in anti-LIN28A-immunoprecipitated fractions from HK-2 and HEK-293T cells was analyzed by qRT-PCR (n=3). **(C)** LIN28A was pulled down by the MALAT1 sense probe (n=3). **(D)** MALAT1 stability in HK-2 cells in response to actinomycin D (n=4). *P < 0.05, **P < 0.01 and ***P < 0.001.

HK-2 cells were transfected with si-LIN28A or si-LIN28A in combination with oe-LIN28A. We found that knockdown of LIN28A reduced the abundance of MALAT1 in HK-2 cells, and simultaneous overexpression of LIN28A restored the expression of MALAT1, suggesting that LIN28A maintained MALAT1 expression in HK-2 cells (**Figure 3A**). RIP assays showed that MALAT1 was efficiently enriched in the anti-LIN28A-immunoprecipitated fractions from HK-2 and HEK-293T cells (**Figure 3B**). Besides, LIN28A was pulled down by the biotinylated MALAT1 probe (**Figure 3C**). These data indicated that MALAT1 directly interacted with LIN28A in HK-2 and HEK-293T cells. As LIN28 functions as an RNA binding protein (RBP) to stabilize target RNAs (23), we hypothesized that LIN28A might regulate MALAT1. To validate the hypothesis, HK-2 cells transfected with si-LIN28A or si-LIN28A in combination with oe-LIN28A were treated with actinomycin D. Silencing of LIN28A impaired MALAT1 stability with a half-life of ~5 hours in HK-2 cells, and simultaneous overexpression of LIN28A restored MALAT1 stability (**Figure 3D**), implying that LIN28A interacted with and stabilized MALAT1.

### MALAT1-Mediated Aggravation of HG-Induced Injury was Dependent on LIN28A

**Figure 4:**
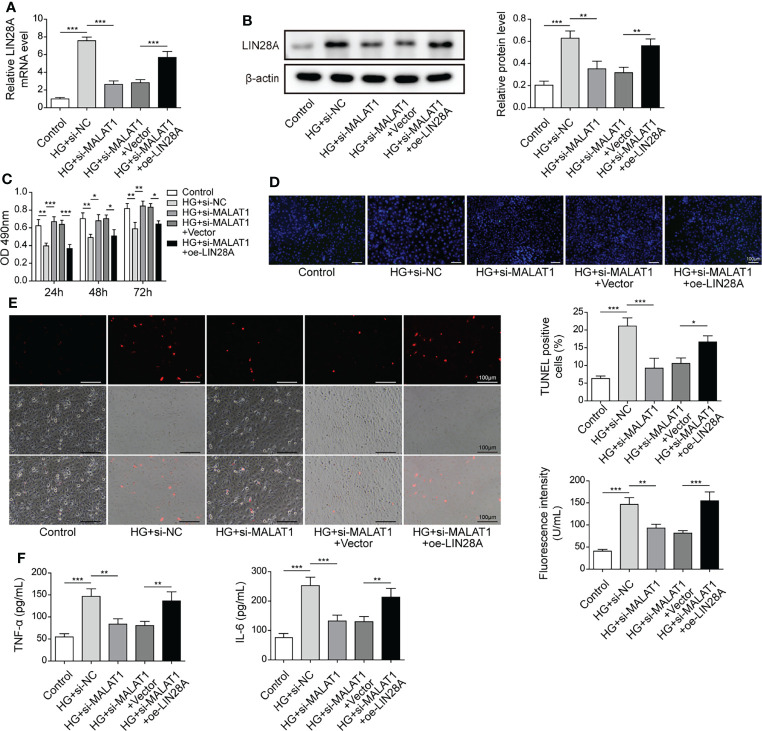
MALAT1-mediated aggravation of HG-induced injury was dependent on LIN28A. HK-2 cells were transfected with si-NC, si-MALAT1, si-MALAT1 + vector or si-MALAT1 + oe-LIN28A and subsequently treated with HG for 48 h **(A)** qRT-PCR analysis of LIN28A (n=4). **(B)** Protein levels of LIN28A were assessed by western blotting (n=4). **(C)** Cell viability was examined by MTT assays (n=4). **(D)** TUNEL staining (TUNEL, green. Scale bar=100 µM) were examined. **(E)** Cellular ROS levels were examined by a fluorometric ROS sensor (red, n=4. Scale bar=100 µM). **(F)** Secretion of TNF-α and IL-6 into culture supernatants (n=4). *P < 0.05, **P < 0.01 and ***P < 0.001.

HK-2 cells were transfected with si-MALAT1 or si-MALAT1 in combination with oe-LIN28A and subsequently treated with HG. The expression of LIN28A were suppressed by si-MALAT1 transfection but enhanced by simultaneous overexpression of LIN28A (**Figures 4A, B**). Knockdown of MALAT1 improved the viability of HG-treated HK-2 cells, but it was abrogated by LIN28A overexpression (**Figure 4C**). Furthermore, knockdown of MALAT1-mediated alleviation of cell apoptosis, ROS generation and secretion of TNF-α and IL-6 were reversed by overexpression of LIN28A (**Figures 4D–F**). Our findings suggested that MALAT1-mediated aggravation of HG-induced injury was dependent on LIN28A in HK-2 cells.

### MALAT1 Facilitated the Interaction of LIN28A and Nox4 to Stabilize Nox4 mRNA in HK-2 Cells

**Figure 5:**
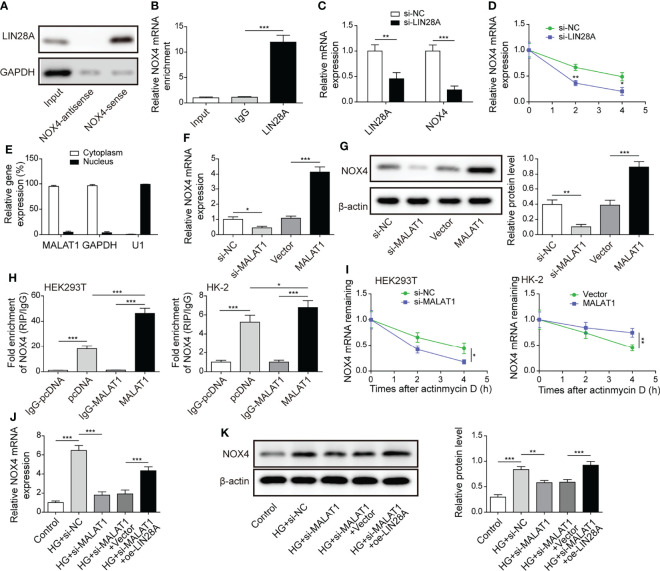
MALAT1 facilitated the interaction of LIN28A and Nox4 to stabilize Nox4 mRNA in HK-2 cells. **(A)** LIN28A was pulled down by the Nox4 sense probe (n=3). **(B)** The enrichment of Nox4 in anti-LIN28A-immunoprecipitated fractions from HK-2 cells was analyzed by qRT-PCR (n=3). **(C)** Levels of LIN28A and Nox4 mRNA in HK-2 cells transfected with si-NC or si-LIN28A (n=4). **(D)** Nox4 mRNA stability in response to actinomycin D was determined by qRT-PCR (n=3). **(E)** The abundance of MALAT1, GAPDH and U1 snRNA in cytoplastic and nuclear fractions (n=4). The expression of Nox4 was analyzed by qRT-PCR (F, n=4) and western blotting (**G**, n=4). **(H)** RIP assays for analyzing the interaction between LIN28A and Nox4 in HK-2 and HEK-293T cells (n=3). **(I)** Nox4 mRNA stability in response to actinomycin D was determined by qRT-PCR in HK-2 and HEK-293T cells (n=3). HK-2 cells were transfected with si-NC, si-MALAT1, si-MALAT1 + vector or si-MALAT1 + oe-LIN28A and subsequently treated with HG for 48 h The expression of Nox4 was determined by qRT-PCR (**J**, n=4) and western blotting (**K**, n=4). *P < 0.05, **P < 0.01 and ***P < 0.001.

As ROS-generating NADPH oxidase Nox4 serves key roles in DN, we examined whether LIN28A interacted with Nox4 in HK-2 cells. RNA pull-down assays showed that LIN28A was pulled down by the Nox4-sense probe, but not by the Nox4-antisense probe (**Figure 5A**). In addition, we found that Nox4 was enriched in the anti-LIN28A-immunoprecipitated fractions from HK-2 cells (**Figure 5B**). HK-2 cells transfected with si-LIN28A showed reduced expression of LIN28A and Nox4 (**Figure 5C**). Furthermore, knockdown of LIN28A reduced the stability of Nox4 mRNA in HK-2 cells in response to actinomycin D (**Figure 5D**). These observations suggested that LIN28A targeted Nox4 to maintain its stability in HK-2 cells. Subsequently, we investigated whether MALAT1 was implicated in the interaction between LIN28A and Nox4. As same as GAPDH, MALAT1 was primarily localized in the cytoplasm of HK-2 cells (**Figure 5E**). Besides, Nox4 expression was inhibited by knockdown of MALAT1 and increased by overexpression of MALAT1 (**Figures 5F, G**). RIP assays showed that Nox4 mRNA was enriched by anti-LIN28A in HEK-293T and HK-2 cells, and overexpression of MALAT1 significantly increased the enrichment (**Figure 5H**). Knockdown of MALAT1 reduced Nox4 mRNA stability, whereas overexpression of MALAT1 increased its stability in HEK-293T and HK-2 cells in response to actinomycin D (**Figure 5I**). HG-induced expression of Nox4 was suppressed by knockdown of MALAT1, but it was partially reversed by simultaneous overexpression of LIN28A in HK-2 cells (**Figures 5J, K**). To conclude, MALAT1 promoted the interaction of LIN28A and Nox4 to maintain Nox4 mRNA stability in HK-2 cells.

### Nox4 Exacerbated HG-Induced HK-2 Cell Injury

**Figure 6:**
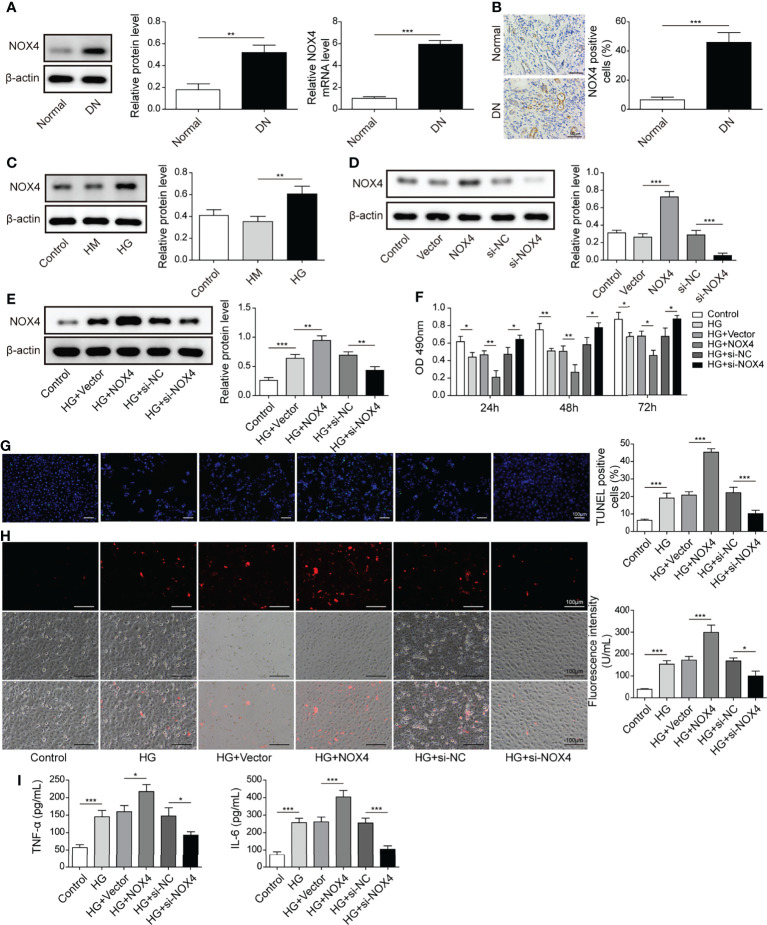
Nox4 exacerbated HG-induced injury in HK-2 cells. **(A)** The expression of Nox4 in DN and normal tissues was analyzed by qRT-PCR and western blotting (n=6). **(B)** IHC staining of Nox4 (n=6). **(C)** Protein levels of Nox4 in HK-2 cells treated with NG, HM or HG were examined by western blotting (n=4). **(D)** Western blotting analysis of Nox4 in HK-2 cells transfected with vector, oe-Nox4, si-NC or si-Nox4 (n=4). HK-2 cells were transfected with vector, oe-Nox4, si-NC or si-Nox4 and subsequently treated with HG for 48 h **(E)** Nox4 was detected using western blotting (n=4). **(F)** Cell viability was examined by MTT assays (n=4). **(G)** TUNEL staining (TUNEL, green. Scale bar=100 µM) were examined. **(H)** Cellular ROS levels were examined by a fluorometric ROS sensor (red, n=4. Scale bar=100 µM). **(I)** Secretion of TNF-α and IL-6 into culture supernatants (n=4). *P < 0.05, **P < 0.01 and ***P < 0.001.

Nox4 was upregulated, and more Nox4 positive cells were observed in DN tissues (**Figures 6A, B**). Besides, Nox4 was upregulated in HK-2 cells treated with HG (**Figure 6C**). Moreover, Nox4 was upregulated in cells transfected with oe-Nox4 and downregulated in cells transfected with si-Nox4 (**Figure 6D**). HG-induced Nox4 expression was significantly increased in HK-2 cells transfected with oe-Nox4 but inhibited in cells transfected with si-Nox4 (**Figure 6E**). Importantly, overexpression of Nox4 further reduced HK-2 cell viability in response to HG, whereas knockdown of Nox4 maintained cell viability (**Figure 6F**). Additionally, HG-induced cell apoptosis, ROS generation and secretion of TNF-α and IL-6 were considerably increased by overexpression of Nox4 but reduced by knockdown of Nox4 (**Figures 6G–I**). These data demonstrated that Nox4 enhanced HG-induced injury in HK-2 cells.

### LIN28A Aggravated HG-Induced Injury by Targeting Nox4

**Figure 7:**
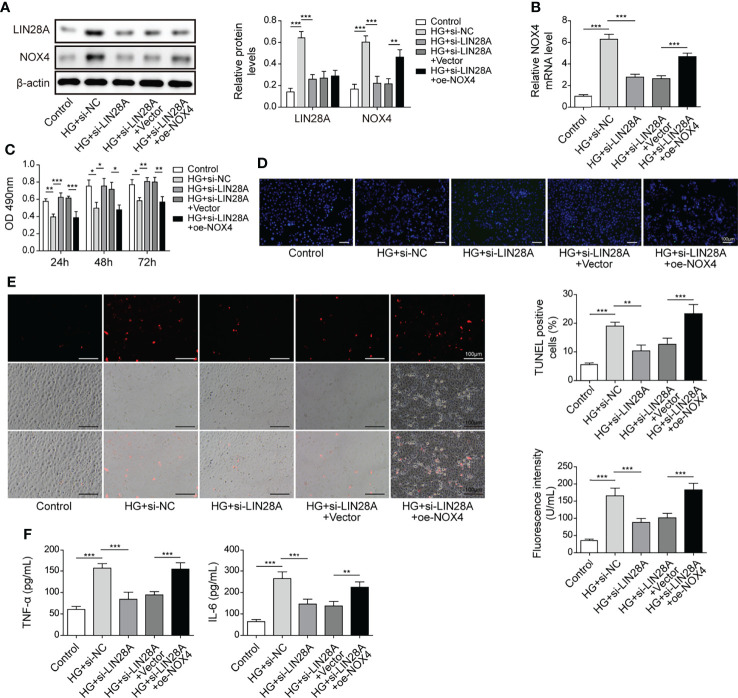
LIN28A aggravated HG-induced injury by targeting Nox4. HK-2 cells were transfected with si-NC, si-LIN28A, si-LIN28A + vector or si-LIN28A + oe-Nox4 and subsequently treated with HG for 48 h **(A)** Western blotting analysis of LIN28A and Nox4 (n=4). **(B)** qRT-PCR analysis of Nox4 (n=4). **(C)** Cell viability was examined by MTT assays (n=4). **(D)** TUNEL staining (TUNEL, green. Scale bar=100 µM) were examined. **(E)** Cellular ROS levels were examined by a fluorometric ROS sensor (red, n=4. Scale bar=100 µM). **(F)** Secretion of TNF-α and IL-6 into culture supernatants (n=4). *P < 0.05, **P < 0.01 and ***P < 0.001.

HK-2 cells were transfected with si-LIN28A or si-LIN28A in combination with oe-Nox4 and treated with HG. HG treatment increased the expression of LIN28A and Nox4, which was suppressed by transfection of si-LIN28A, and Nox4 expression was increased by transfection of oe-Nox4 (**Figures 7A, B**). Knockdown of LIN28A improved HG-treated HK-2 cell viability, but simultaneous overexpression of Nox4 abolished this effect (**Figure 7C**). Intriguingly, silencing of LIN28A mitigated cell apoptosis, ROS generation and secretion of TNF-α and IL-6 in HG-treated HK-2 cells, which were largely abrogated by Nox4 overexpression (**Figures 7D–F**). These data demonstrated that LIN28A targeted Nox4 to aggravate HG-induced injury in HK-2 cells.

### Knockdown of Nox4 Relieved HG-Induced Injury by Suppressing the AMPK/mTOR Signaling in HK-2 Cells

**Figure 8:**
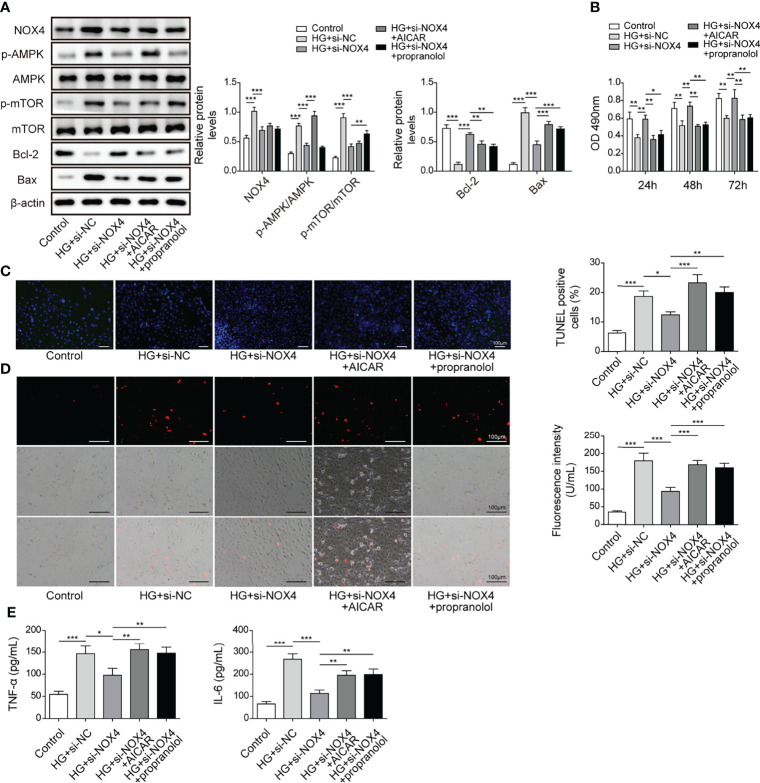
Knockdown of Nox4 relieved HG-induced injury by suppressing the AMPK/mTOR signaling. HK-2 cells were transfected with si-Nox4 and treated with HG, HG + AICAR or HG + propranolol. **(A)** Protein levels of Nox4, p-AMPK, AMPK, p-mTOR, mTOR, Bcl-2 and Bax (n=4). **(B)** Cell viability was examined by MTT assays (n=4). **(C)** TUNEL staining (TUNEL, green. Scale bar=100 µM) were examined. The nucleus was stained with DAPI (blue). **(D)** Cellular ROS levels were examined by a fluorometric ROS sensor (red, n=4.Scale bar=100 µM). **(E)** Secretion of TNF-α and IL-6 (n=4) into culture supernatants. *P < 0.05, **P < 0.01 and ***P < 0.001.

As Nox4/AMPK/mTOR signaling has been reported to be implicated in diabetic kidney disease (21), we hypothesized that the AMPK/mTOR signaling might be implicated in Nox4-mediated regulation of HG-induced injury. HK-2 cells were transfected with si-Nox4 and treated with HG in combination with the AMPK activator AICAR or the mTOR activator propranolol. HG treatment increased the expression of Nox4 and Bax and phosphorylation of AMPK (p-AMPK) and mTOR (p-mTOR) and reduced Bcl-2 expression in HK-2 cells, which were largely abolished by knockdown of Nox4 (**Figure 8A**). AICAR and propranolol reversed Nox4 knockdown-mediated regulation of p-AMPK and p-mTOR and expression of Bax and Bcl-2 in HG-treated HK-2 cells (**Figure 8A**). Moreover, knockdown of Nox4 increased the viability of HG-treated HK-2 cells, but it was reversed by AICAR or propranolol treatment (**Figure 8B**). Besides, knockdown of Nox4-mediated alleviative effects on HG-induced cell apoptosis, ROS generation and secretion of TNF-α and IL-6 were abolished by AICAR or propranolol (**Figures 8C–E**). Our findings indicated that knockdown of Nox4 attenuated HG-induced injury through suppression of the AMPK/mTOR signaling in HK-2 cells.

### Knockdown of MALAT1 Alleviated Renal Tubular Epithelial Injury by Suppressing LIN28A and the Nox4/AMPK/TOR Signaling Axis in Rats With DN

**Figure 9:**
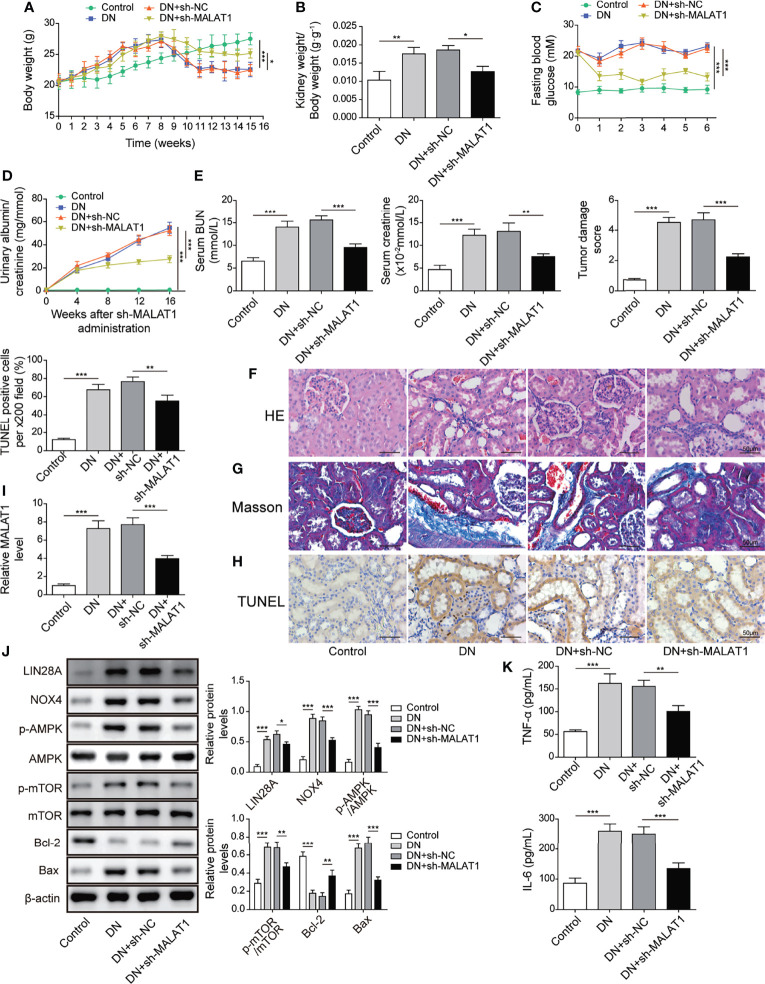
Knockdown of MALAT1 alleviated renal tubular injury by suppressing LIN28A and the Nox4/AMPK/TOR signaling axis in rats with DN. SD rats with DN were intravenously injected with shMALAT1 lentiviral particles and sequentially fed a high glucose and fat diet for 5 months. **(A)** Body weight of rats (n=6 each group). **(B)** Kidney to body weight ratio (n=6 each group). **(C)** Fasting blood glucose (n=6 each group). **(D)** Urinary albumin to creatinine ratio (n=6 each group). **(E)** The concentration of BUN and creatinine in serum (n=6 each group). **(F)** H&E staining of kidney sections (Scale bar=50 µM). **(G)** Masson’s Trichrome staining of kidney sections (Scale bar=50 µM). **(H)** TUNEL staining for cell apoptosis analysis in kidney sections (Scale bar=50 µM). **(I)** qRT-PCR analysis of MALAT1 in the kidneys (n=6). **(J)** Protein levels of LIN28A, Nox4, p-AMPK, AMPK, p-mTOR, mTOR, Bcl-2 and Bax in the kidneys (n=6). **(K)** The concentrations of TNF-α and IL-6 in serum (n=6). *P < 0.05, **P < 0.01 and ***P < 0.001.

A rat model of DN was established. Subsequently, rats with DN were intravenously injected with shMALAT1 or sh-NC lentiviral particles and fed a high glucose and fat diet for 5 months. Rats with DN showed ascending body weight in the first 8 weeks and declining body weight after 8 weeks, but the degree of weight loss was obviously slowed down in rats injected with shMALAT1 (**Figure 9A**). Similarly, elevated kidney weight (KW)/body weight (BW), fasting blood glucose, albumin-to-creatinine ratio (ACR), blood urea nitrogen (BUN) and creatinine were observed in rats with DN, whereas all of them were significantly reduced by shMALAT1 injection (**Figures 9B–E**). Furthermore, we observed severe renal tubule damage and increased collagen deposition and cell apoptosis in renal tubules, and injection of shMALAT1 markedly attenuated these manifestations (**Figures 9F–H**). The expression of MALAT1 was dramatically promoted in DN rats and greatly reduced by injection of shMALAT1 (**Figure 9I**). These observations demonstrated that knockdown of MALAT1 alleviated renal tubular epithelial injury in DN rats. Elevated levels of LIN28A, Nox4, p-AMPK and p-mTOR and Bax and reduced Bcl-2 expression in DN rats were largely reversed by knockdown of MALAT1 (**Figure 9J**). Increased secretion of TNF-α and IL-6 was also significantly inhibited by knockdown of MALAT1 in DN rats (**Figure 9K**). Collectively, these results implied that knockdown of MALAT1 alleviated renal tubular epithelial injury through suppression of LIN28A and the Nox4/AMPK/TOR signaling axis in DN.

## Discussion

Approximately 40% of patients with diabetes will have DN and the prevalence is still increasing (24, 25). DN impairs kidney function and leads to kidney failure, increasing the mortality of diabetic patients (26, 27). Here, we reported that MALAT1, LIN28A and Nox4 were upregulated in DN, and their overexpression significantly promoted apoptosis, oxidative stress and inflammatory cytokine secretion and impaired cell viability. MALAT1 interacted with LIN28A and contributed to stabilizing Nox4 mRNA. Furthermore, MALAT1 exacerbated renal tubular injury through interacting with LIN28A and stabilizing Nox4. In addition, the Nox4/AMPK/mTOR signaling was implicated in MALAT1-mediated exacerbation of renal tubular injury. Taken together, we demonstrated that MALAT1 aggravated HG-induced renal tubular epithelial cell injury by interacting with LIN28A and activating the Nox4/AMPK/mTOR signaling in DN for the first time.

MALAT1 is a key regulator in diabetic kidney disease. Li et al. found that MALAT1 enhanced pyroptosis *via* reducing miR-23c expression and increasing the expression of ELAVL1 and NLRP3 (28). Moreover, MALAT1 promoted epithelial-to-mesenchymal transition through activation of the Wnt/β-catenin signaling (29). Circulating MALAT1 in the blood from DN patients were significantly elevated, which could be used as a diagnostic biomarker for DN in diabetic patients (30). Growing evidence has indicated that MALAT1 can be a potential diagnostic and therapeutic targets for diabetic renal diseases (31). Consistently, we reported that MALAT1 was upregulated in DN tissues and HG-treated HK-2 cells, and MALAT1 aggravated HG-induced renal tubular injury, further indicating that MALAT1 could be a therapeutic target for inhibiting DN progression. Furthermore, we also observed highly expressed LIN28A, suggesting the interaction between MALAT1 and LIN28A in DN. LIN28A has been reported to stabilize lncRNAs such as SNHG14 and FBXL19-AS1 (32, 33). As expected, MALAT1 interacted with LIN28A and stabilized by LIN28A in HK-2 cells, identifying a novel linkage between MALAT1 and LIN28A in DN. We also demonstrated that MALAT1-mediated aggravation of HG-induced HK-2 cell injury was dependent on LIN28A for the first time.

We observed that Nox4 was upregulated in DN tissues and HG-treated HK-2 cells. Nox4 has been demonstrated to serve key roles in regulating DN progression. The regulation of epithelial Na+ channel in DN was dependent on Nox4 (34). Ilatovskaya and colleagues reported that the Nox4/TRPC6 pathway was implicated in the regulation of podocyte calcium and kidney injury in DN (35). In this study, we demonstrated that elevated Nox4 expression exacerbated HG-induced renal tubular epithelial cell injury. Our data and previous studies in diabetic conditions suggest therapeutic potential of Nox4 inhibitors for DN treatment (36, 37). As LIN28A functions as an RNA binding protein and stabilizes target mRNAs (23, 38), we examined the association of MALAT1, LIN28A and Nox4 and found that MALAT1 facilitated the interaction of LIN28A and Nox4 to stabilize Nox4 mRNA in HK-2 cells. In addition, LIN28A-mediated regulation of HG-induced HK-2 cell injury was dependent on Nox4. The AMPK/mTOR signaling was found to be regulated by Nox4 and implicated in DN. The AMPK/mTOR signaling is a key signaling in autophagy, and DN could be alleviated through AMPK/mTOR-mediated activation of autophagy (39, 40). The involvement of AMPK/mTOR-induced autophagy in DN will be investigated.

**Figure 10:**
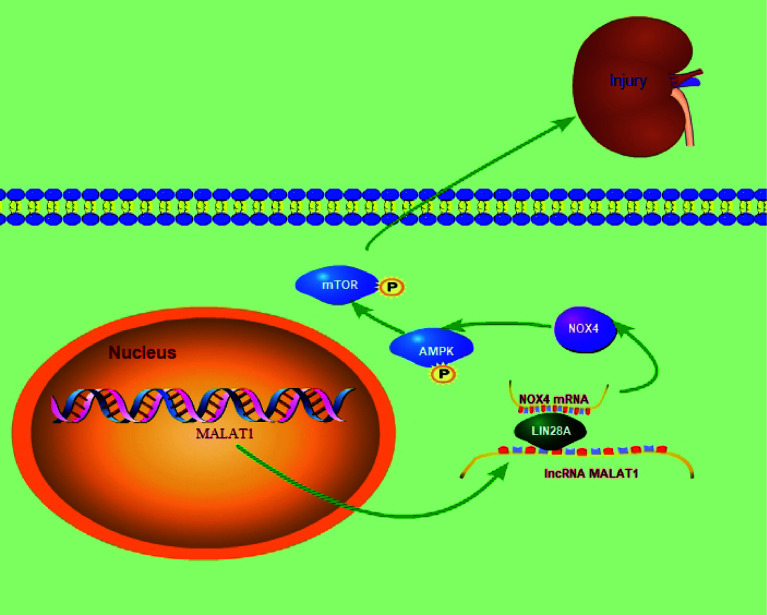
The graphical abstract of the manuscript.

In summary, we demonstrated that MALAT1 promoted the interaction of LIN28A and Nox4 to stabilize Nox4 stability and activating the AMPK/mTOR signaling, thus aggravating HG-induced renal tubular epithelial cell injury in DN, which identified a novel MALAT1/LIN28A/Nox4 axis in DN progression (**Figure 10**). Our findings deepen understanding of DN pathogenesis. In addition, our study supports the notion that MALAT1 promoted DN and indicates that MALAT1, LIN28A and Nox4 serve as potential therapeutic targets for restraining DN progression. Further investigations are ongoing to elucidate the nature of MALAT1/LIN28A/Nox4-mediated regulation of DN for clinical application. More clinical samples and *in vivo* assays in animal models of DN should be adopted in future investigations. As lncRNAs can act as miRNA sponges, studies are ongoing to investigate other potential miRNA targets of MALAT1 and associated signaling pathways in the context of DN.

## Data Availability Statement

The original contributions presented in the study are included in the article/supplementary material. Further inquiries can be directed to the corresponding author.

## Ethics Statement

The studies involving human participants were reviewed and approved by The Medical Ethics Committee of Central South University. The patients/participants provided their written informed consent to participate in this study. The animal study was reviewed and approved by The Medical Ethics Committee of Central South University.

## Author Contributions

PS designed the study, analyzed the data, interpreted the results, and drafted the manuscript. YC, ZL, LX, HL, JW, and LS contributed to the data collection and manuscript revision. LH was the corresponding author and was involved in the study design, data interpretation, and manuscript revision. All authors contributed to the article and approved the submitted version.

## Funding

This work was supported by Hunan Provincial Natural Science Foundation for Outstanding Youth (No. 2022JJ10093, 2020JJ2020), Major Research and Development Program of Hunan Province (2020SK2116), National Natural Science Foundation of China (No. 81870500, 81800649 ,82160135), Hunan Provincial Clinical Medical Technology Innovation Guide Project (2020SK53402), China International Medical Foundation (Z-2017-24-2037), Research Project from Blood Purification Center Branch of Chinese Hospital Association in 2021 (CHABP2021-12), Hainan key research and development projects (ZDYF2022SHFZ016).

## Conflict of Interest

The authors declare that the research was conducted in the absence of any commercial or financial relationships that could be construed as a potential conflict of interest.

## Publisher’s Note

All claims expressed in this article are solely those of the authors and do not necessarily represent those of their affiliated organizations, or those of the publisher, the editors and the reviewers. Any product that may be evaluated in this article, or claim that may be made by its manufacturer, is not guaranteed or endorsed by the publisher.
